# Chromosomal Aberrations in Induced Pluripotent Stem Cells: Identification of Breakpoints in the Large *DCC* Gene and *HIST2* Histone Gene Cluster

**DOI:** 10.3390/ijms26167728

**Published:** 2025-08-10

**Authors:** Diana Zheglo, Victoria O. Pozhitnova, Anastasiia V. Kislova, Zhanna G. Markova, Danila Kiselev, Philipp S. Sviridov, Valeria Sviridova, Lyajsan I. Gumerova, Svetlana A. Smirnikhina, Almaqdad Alsalloum, Svetlana V. Pylina, Sergey Ivanovich Kutsev, Ekaterina Sergeevna Voronina

**Affiliations:** 1 Research Centre for Medical Genetics, Moskvorechie 1, Moscow 115478, Russia; 2 Federal Research Center for Innovator and Emerging Biomedical and Pharmaceutical Technologies, Baltiyskaya St. 8, Moscow 125315, Russia; 3 Endocrinology Research Centre, Moscow 115478, Russia

**Keywords:** induced pluripotent stem cells, karyotype, 1q gain, *DCC*, histone, breakpoint, genome instability, replication stress, common fragile sites

## Abstract

Genome instability in induced pluripotent stem cells (IPSC) poses a significant challenge for their use in research and medicine. Cataloging and precisely describing all the identified aberrations that arise during cell reprogramming, expansion, and differentiation is essential for improving approaches to instability prevention and ensuring genetic quality control. We report the karyotypic analysis of 65 cell lines derived from skin fibroblasts, urinal sediment, and peripheral blood mononuclear cells of 33 individuals, 82% of whom suffer from monogenic genetic disorders not associated with genetic instability. Trisomy of chromosomes 20 and 8 was revealed recurrently, while the 1q arm was the most frequently affected region involved in interstitial duplications and unbalanced translocations with chromosomes 15 and 18. The localization of rearrangement breakpoints identified by SNP arrays within the large *DCC* gene and histone gene clusters links genetic instability in IPSCs to replication-stress-induced chromosome breakage at common and early replicating fragile sites.

## 1. Introduction

Induced pluripotent stem cells (IPSCs) derived from the cells of patients with genetic disorders are an important tool for studying disease mechanisms and developing personalized therapy. Their ability to differentiate into nearly any tissue type and unlimited proliferation capacity allows researchers to access histologically and genetically relevant models that reflect the characteristics of the patients’ original cells [1]. However, the cellular stress associated with reprogramming and in vitro expansion, along with pluripotency-specific low mitotic fidelity [2], relaxed checkpoint control [3,4], and high basal levels of replication stress [5,6], predisposes IPSCs to mosaic genetic alterations. Subsequent culture adaptation facilitates the accumulation of specific aberrations and shapes the landscape of recurrent chromosomal rearrangements in IPSCs. Culture-acquired genetic alterations can impact critical cell properties, including differentiation capacity, pluripotency maintenance, and sensitivity to apoptosis [7,8,9,10]. Therefore, these changes must be detected and eliminated promptly.

In the largest reported IPSC collections, karyotype abnormalities have been detected in 22–23% of analyzed samples [8], while prolonged passaging can result in up to 80% of lines exhibiting aberrations [11]. Recurrent aberrations can account for more than 90% of all genetic alterations and can be used for targeted assessment of genetic stability of cell lines [12,13]. The list of recurrent chromosomal rearrangements reproduced in the majority of studies includes gains of chromosomes or chromosomal segments 20/20q, 1q, 12, and 17, less common gains of X and 8, and losses of chromosomes 10, 18, and 22. However, some reports indicate alternate instability patterns in IPSCs, which could originate from genetic diversity of the samples or variations in laboratory techniques [14,15]. Additionally, the recurrence of gains at 1q and 20q has increased in recent years compared to earlier studies, reflecting the evolution of culturing protocols [8]. The 1q gain has been associated with feeder-free protocols and high-density cell culture [8,16], while the 20q gain favors survival after single-cell passaging [17]. Therefore, accurate cataloging and characterization of all culture-acquired genetic alterations in IPSCs are essential for implementing the most relevant techniques to monitor the genetic stability of IPSC lines. Furthermore, identifying the most vulnerable genomic regions in IPSCs can provide insight into the mechanisms of instability and the functional impact of genetic alterations.

We report the karyotyping of 65 cell lines, complemented by SNP-array analysis of 1q structural rearrangements. The pattern and frequency of the identified abnormalities align with those previously described by the Stem Cell Consortium. The localization of breakpoint junctions within the large neuronal and tumor suppressor gene *DCC* and the HIST2 histone gene cluster supports the proposed role of chromosome breakage at common and early-replicating fragile sites in contributing to genomic instability in IPSCs.

## 2. Results

### 2.1. Sample Description

The study involved 65 IPSC lines, which were obtained in four laboratories and karyotyped by our facility. The lines were obtained from somatic cells of 33 individuals reprogrammed with the integrating method (Lentivirus, 3 lines) and non-integrating methods (RNA replicon vector, 3 lines, and Sendai virus, 59 (91%) lines). Parental cells were dermal fibroblasts (26 donors, 54 lines), peripheral blood mononuclear cells (4 donors, 5 lines), or urinal sediment cells (3 donors, 6 lines). Most of the donors (27/33, 82%) were affected carriers of monogenic diseases (16 nosologies listed in Table 1); the remaining six (18%) donors were unaffected mutation carriers or individuals without genetic diagnoses. Detailed information about the cell lines is provided in Supplementary Table S1.

Different lines (clones) from the same donor represented the progeny of the individual colonies separated at the first passage after reprogramming. Karyotyping was performed at the time of culture characterization (passages 5 to 40, median passage is 17), with six lines being reanalyzed at later passages, and two lines karyotyped after gene editing.

### 2.2. Incidence of Karyotype Abnormalities

Karyotype abnormalities were identified in 16 out of 70 analyses (23%). The frequency of abnormal cell lines with clonally unique aberrations was 21% (13 out of 62 cell lines), which included five lines exhibiting both normal and abnormal karyotypes at different passages. In cell lines derived from healthy donors, the frequency of clonally unique aberrations in the initial analysis was 2 out of 10, which increased to 4 out of 10 after re-karyotyping at later passages. Among the 52 cell lines from patients suffering genetic disorders, 9 unique lines (17.3%) exhibited chromosomal aberrations. 

In most cases, only a single aberrant cell line was identified per donor, with the other lines from the same donor being genetically intact. However, in the lines from three donors, we observed two genetically altered lines. In all three cases, the chromosomal aberrations were identical for the two lines from one donor, so we considered these lines to have a common clonal origin. In samples from one cystic fibrosis patient, two distinct clonal aberrations were identified: a line carrying structural rearrangement of the 1q chromosome and a line exhibiting elevated chromatid breakage. It remains uncertain whether the clone with the rearranged 1q chromosome originated from the cells with fragile chromosomes. In a cell line derived from fibroblasts of a non-affected carrier of a recessive disease allele, two independent clones emerged consecutively at different passages, featuring a 1q duplication and a pseudoisodicentric chromosome 20. These changes can be attributed to a bottleneck effect that occurred during the cultivation process between the two karyotype analyses.

When analyzing the tissue source of the reprogrammed cells, 1 of 6 urinal-sediment-derived lines, 1 of 4 mononuclear-cell-derived lines, and 11 of 52 fibroblast-derived lines (21%) acquired genetic changes. These results do not indicate any cell-type-specific or donor-diagnosis-specific predisposition to instability in our sample. However, larger samples are needed for more accurate conclusions. 

### 2.3. Recurrent and Non-Recurrent Aberrations

**Figure 1:**
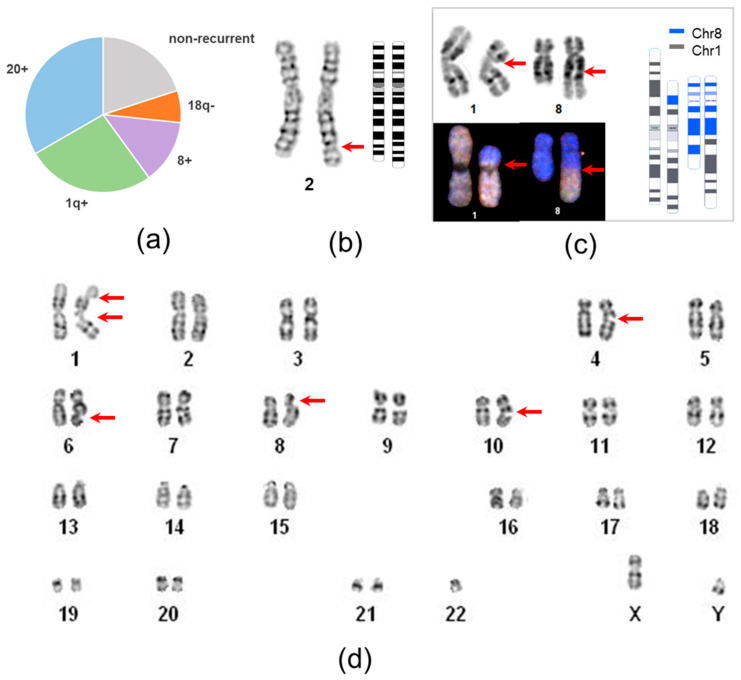
Impact of different types of karyotype abnormalities on iPSC genetic instability and structure of non-recurrent aberrations. (**a**) Proportions of recurrent (chr20 gain, 1q gain, 8 gain, 18q loss) and non-recurrent genetic alterations; (**b**) G-banding image of dup(2)(q32;q33) in the P8L1 cell line; (**c**) G-banding image and spectral karyotyping paint of a balanced t(1;8)(p21;q22) in the KTV cell line. Schematic ideogram was generated using the CyDAS program; (**d**) karyogram of the P5L5 line with multiple spontaneous chromatid gaps and breaks. Arrows indicate breakpoints of aberrations.

Spontaneous genetic changes in iPSCs undergo selection in vitro. The well-recognizable group of “recurrent” karyotype abnormalities, including whole and partial gains of chromosomes 20, 1, 12, 17, 8, and X and losses of chromosomes 10 and 18, changes cell phenotype and outcompetes wild-type cells. Our cell line karyotyping revealed 14 aberrant cell clones, and 11 (78.6%) of the aberrations were classified as recurrent (Figure 1a and Table 2).

### 2.4. Structural Characterization of Recurrent Chromosomal Rearrangements

**Figure 2:**
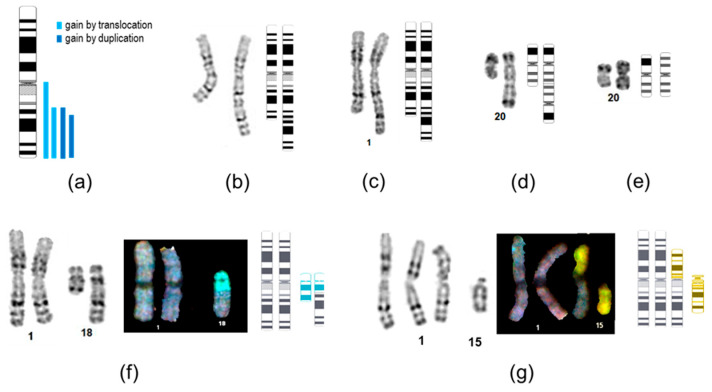
Structure of recurrent chromosomal rearrangements. (**a**) Chromosomal localization of 1q gains. (**b**–**e**) G-banded images of rearranged chromosomes and respective ideograms of lines: (**b**) P5Lmix, dup(1)(q23q44); (**c**) P16L2, dup(1)(q25q44), (**d**) P16L2, psu idic(20), and (**e**) MNA cl3, i(20q). (**f**,**g**) G-banding, spectral karyotyping, and graphical illustration of translocations in HAS cl38 and MAK-F lines. Ideograms were generated using the CyDAS web interface, the color code of ideograms corresponds to SKY staining of respective chromosomes.

The most prevalent genetic alteration observed was the gain of 20q, which manifested as trisomy 20 (*n* = 3), isochromosome 20q, or pseudoisodicentric chromosome (Figure 2d,e). Notably, small duplications or amplifications at 20q11.21 were not detected, as they are beyond the resolution of karyotyping.

Chromosome 1 was involved in five structural rearrangements, four of which resulted in 1q arm gain. Consistent with previous reports, two of these gains were organized as interstitial duplications, with proximal breakpoints located at q23 and q25 according to G-banding, lines P5Lmix and P16L2, respectively. The DNA of the P16L2 line was available for SNP-array analysis, so the 1q duplication breakpoints were re-mapped to q23.3–q42.12 (chr1:161,007,698–226,095,452, hg38). These breakpoints were located within the *F11R* gene encoding junctional adhesion molecule A, which is a cell surface molecule expressed by IPSCs [18], and at an intergenic region adjacent to the *H3-3A* histone gene, respectively (Supplementary Figure S1).

The other two 1q gains resulted from translocations with chromosomes 18 and 15. In the HAS cell line, the 1q breakpoint was mapped by G-banding to q23, resulting in a derivative chromosome that retained the centromere of chromosome 18 while losing a distal part of 18q (Figure 2f). Subsequent SNP array analysis (Supplementary Figure S2) refined the breakpoint at 1q to the q21.2 band (chr1:149,817,456 hg38) within the *H4C14* histone gene of the replication-dependent histone cluster. This region is enriched in segmental duplications and pseudogenes, predisposing to instability and low SNP-array probe density. The breakpoint on chromosome 18 was located at 18q21.2 (chr18:52,905,053), within the large *DCC* gene that encodes the neuronal receptor for netrin and pro-apoptotic tumor suppressor gene. In the MAK-F cell line, the presence of centromere 1 on the derivative chromosome was confirmed by FISH analysis. The low clonality of the rearrangement (11% as determined by FISH analysis of 1000 nuclei) did not allow for the resolution of the breakpoints using SNP arrays.

### 2.5. Dynamics of Abnormal Cell Clones Under Passaging

Genetic alterations in IPSCs provide a substrate for both positive and negative selection in vitro and tend to accumulate with extended cultivation time. Chromosomal aberrations were observed in cell passages from 6 to 39, while other cell lines maintained a stable karyotype from passages 5 to 40. For several cell lines enrolled in other experiments (the exact number is unspecified), karyotyping was performed at different passages, and discordant karyotyping results were obtained for five of those lines (Supplementary Table S1). Two non-recurrent aberrations, chromatid breaks and dup2q, were detected at first analysis (12 and 16 passages, respectively), while at later passages, only a normal clone was present. Recurrent trisomy and isodicentric chromosome 20 were detected at second analysis (passages 24–27) and can be classified as culture-acquired aberrations.

Trisomy 8 has been documented as a recurrent aberration in a subset of studies. In our sample, karyotyping revealed low-level (5.6% and 10%) trisomy 8 in two unrelated lines. Given that the detection threshold of standard karyotyping is approximately 10% [12], we conducted a centromeric FISH analysis on the same cell suspensions for the P19L7 line (3/30 abnormal metaphases), the autologous P19L3 line (0/30 abnormal metaphases), and their parental urinal sediment cells, USP19. Unexpectedly, analysis of 1000 nuclei indicated the presence of 7.3%, 10.9%, and 2.9% trisomic cells in P19L7, P19L3, and USP19, respectively. The level of trisomy for autosomes in karyotypically normal cell lines was estimated at 4.0% ± 1.3% (mean ± SD), suggesting that both iPSC lines were close to borderline values, with the trisomy level in P19L3 being three times the standard deviation above the mean.

To further elucidate the instability of P19L3, we cultured the cells for an additional 10 passages (from passage 18 to 28). FISH analysis at this stage revealed 6.4% trisomic nuclei, suggesting that the observed abnormality is not a culture-favoring condition but rather indicates sporadic fluctuations in the abnormal clone fraction.

## 3. Discussion

Most recurrent karyotypic abnormalities in IPSCs are known to displace normal cells over several passages of in vitro cultivation. In our study, low-level mosaicism of trisomy 8 was sustained at a stable level following prolonged cell expansion. The functional impact of trisomy 8 on iPSCs remains largely unexplored. Its frequency has been reported to be approximately 13% of all recurrent abnormalities in the WiCell collection, although it was not identified among the recurrent aneuploidies in the Centre for Stem Cell Biology collection [8]. Asymptomatic tissue-specific mosaicism for trisomy 8 in patients has been reported, with the level of abnormal clone reaching 63% in uncultured blood lymphocytes, 18% in buccal epithelium, and 0% in fibroblasts [19]. This may suggest that an abnormal clone can originate from parental donor tissue, or false-positive centromeric FISH analysis in our study. Nonetheless, further studies are required to define the functional interpretation for chromosome 8 low-level mosaicism in IPSCs.

The 1q arm gains in IPSCs have been reported to occur through interstitial duplications or derivative chromosomes with various translocation partners [20]. The minimal overlapping region encompasses the MDM4 gene at 1q32, and most of the proximal breakpoints are located at the centromeric region, 1q21, 1q23, and 1q25 [20], which aligns with our findings.

There is limited literature regarding the structure of breakpoint junctions in recurrent rearrangements. For the 20q11.21 gain, breakpoints of duplication and triplication have been identified using long-read sequencing, revealing direct tandem repeat orientation and microsatellite motifs at the breakpoint junctions, which suggest microhomology-mediated break-induced replication [21]. To our knowledge, the fine structure of 1q gain and 18q loss breakpoints has not yet been the focus of any studies. In our analysis, two out of three 1q breakpoints identified using SNP arrays were located within the histone gene cluster regions at q21.2 and q42.1. The 18q loss breakpoint was mapped to the large *DCC* gene corresponding to the *FRA18B* common fragile site, which is active in lymphocytes. The same 18q21.2 band has been reported to harbor the 18q loss breakpoints in the VUB04 and VUB13 embryonic stem cell lines [10].

The precise mechanisms underlying the formation of rearrangements in iPSCs are not yet fully understood, and a detailed analysis of their breakpoints could provide insights into these mechanisms. High constitutional levels of replication stress [5,6], along with the localization of CNVs at regions of common fragile sites [22], suggest that chromosomal fragility may play a role in genomic instability of IPSCs. Additionally, exogenous nucleosides have been shown to enhance the genetic stability of IPSC cultures by reducing replication stress [23]. However, the repertoire of pluripotent-cell-specific fragile sites remains largely undefined [24,25]. Our findings support the proposed role of replication stress and *FRA18B* breakage in the genomic instability of IPSCs. In mouse lymphocytes, early-replicating fragile sites have been identified, which present chromosome loci prone to breakage under replication inhibition at early S-phase and are associated with highly transcribed gene clusters, including two histone clusters [26]. Elevated expression of histone genes during the S-phase may lead to transcription–replication collisions and double-strand DNA break formation in IPSCs. However, no evidence of fragility at histone-encoding chromosomal bands in human IPSCs has been reported to date. In summary, comprehensive whole-genome profiling of replication-stress-sensitive genomic regions in IPSCs, including common and early-replicating fragile sites, along with high-resolution analysis of rearrangement breakpoint junctions, will elucidate the mechanisms underlying chromosomal instability in these cells.

## 4. Materials and Methods

### 4.1. Cell Culture

Cells were provided by 4 laboratories, namely the Research Centre for Medical Genetics [27,28,29,30,31,32,33,34,35,36], Mental Health Research Center [37], Moscow Institute of Physics and Technology [38,39], and Endocrinology Research Centre. Cells were cultured at 37 °C and 5% CO_2_ on Matrigel-coated culture plates in TeSR E8 Medium (Stemcell Technologies, Vancouver, BC, Canada), passaged every 4–10 days with Cell Dissociation Reagent, Versen (Pan-Eco, Moscow, Russia), in a ratio split of 1:6 to 1:10 in TeSR E8 Medium with 5 μM Y27632 (Stemcell Technologies).

### 4.2. Karyotyping 

Cells at approximately 70% confluency were arrested by 0.1 μg/mL demecolcine (Sigma-Aldrich, Burlington, MA, USA), harvested by trypsinization, hypotonized 14 min in 0.075M KCl at 37 °C and fixed in ice-cold methanol and acetic acid freshly mixed at the proportion 3:1. Cell suspension was dropped onto precooled, wet slides, allowed to spread for 1 min, and then briefly warmed over the flame of a gas burner. Dried slides were stained with a LumiMount^®^ DAPI Fluorescence Mounting Medium (Lumiprobe, Moscow, Russia), contrasted with 0.3 mg/mL Actinomycin D (Serva, Heidelberg, Germany). Fluorescent images were inverted, and at least 15–20 metaphase images were analyzed using CytoLabView software (ASI, Carlsbad, CA, USA) according to ISCN 2020 nomenclature.

### 4.3. FISH and Spectral Karyotyping (SKY)

For detailed analysis of rearrangement structure or frequency, cytogenetic slides were treated with 250 U/mL pepsin in 0.01N HCL at 37 °C for 5 min, washed in PBS with 50 mM MgCl_2_, postfixed in 1% paraformaldehyde in PBS with 50 mM MgCl_2_ for 10 min., washed in 2xSSC, and dehydrated in ethanol. Probes SE 8 (D8Z1) red (Poseidon, Kreatech, Amsterdam, Netherlands) or SKY paint probe were applied, sealed with rubber cement, co-denatured with specimens, and hybridized in a hybridizer CytoHIB CT500 (Cytotest, Beijing, China) according to the probe manufacturer’s recommendations. Slides were washed in SSC/detergent solutions and counterstained using LumiMount® DAPI Fluorescence Mounting Medium (Lumiprobe). Images were captured using a AxioImager A2 microscope (Zeiss, Oberkochen, Germany) equipped with a SKY detection complex and analyzed using the CytoLabView software (ASI).

### 4.4. SNP Arrays

For analysis of structural rearrangements, cell cultures containing a minimum of 20–30% abnormal clones (measured by karyotyping or FISH) were harvested, and DNA was isolated using standard phenol-chloroform extraction. The CytoScan HD arrays (Affymetrix, Santa Clara, CA, USA.) were applied following the manufacturer’s protocols. Microarray-based copy number analysis was performed using the Chromosome Analysis Suite software version 4.4 (Thermo Fisher Scientific Inc., Waltham, MA, USA).

## Figures and Tables

**Table 1:** Nosologies of cell donors.

Diagnoses	No of Donors	No of Lines
cystic fibrosis	8	16
fibrodysplasia ossificans progressiva	3	6
glycogen storage disease I	2	3
multiple endocrine neoplasia 1	2	6
mucopolysaccharidosis type IVb	1	3
Maroteaux–Lamy syndrome	1	2
Leber’s hereditary optic neuropathy	1	3
Duchenne muscular dystrophy	1	3
Cone dystrophy with supernormal rod response	2	2
maturity-onset diabetes of the young type 3	1	2
maturity-onset diabetes of the young type 10	1	1
maturity-onset diabetes of the young type 12	1	2
X-linked adrenoleukodystrophy	1	1
lysosomal acid lipase deficiency	1	3
schizophrenia (de novo *SLC6A1* variant)	1	1
Subtotal (diseased)	27 (82%)	54 (83%)
Healthy/unaffected carrier	6	11
Total	33	65

**Table 2:** Genetic changes revealed in iPSC lines by karyotyping.

Aberration	Culture-Acquired	Cell Lines	Recurrent Rearrangement Type	Percentage in Present Study
trisomy 20	nd	P4L5	chr20 gain	8.6% of all tests, 38.5% of unique aberrant lines
trisomy 20	yes	P12L3		
trisomy 20	nd	P13L10		
i(20q)	nd	MNA cl3		
i(20q)	nd	MNA e67		
psu idic(20)	yes	P16L2 *		
1q duplication			1q gain	7.2% of all tests, 30.8% of unique aberrant lines
dup(1)(q23q44)	nd	P5Lmix		
dup(1)(q25q44) *	nd	P16L2 **		
1q translocation-duplication				
der(18)t(1;18)(q23;q21.2) ***	nd	HAS cl38	1q gain, 18q loss	
der(18)t(1;18)(q23;q21.2) ***	nd	HAS cl51		
der(1)t(1;15)(p11;q11.2)	yes	MAK-F	1q gain	
trisomy 8	nd	P19L7	chr8 gain	2.9% of all tests, 15.4% of unique aberrant lines
trisomy 8	nd	P22L1		
balanced translocation			non-recurrent	5.8% of all tests, 23.1% of unique aberrant lines
t(1;8)(p21;q22)	nd	KTV cl3		
t(1;8)(p21;q22)	nd	KTV cl11		
dup(2)(q32;q33)	nd	P8L1		
multiple chromatide breaks (chtb)	nd	P5L5		

nd—no data, the rearrangement was present from initial analysis of the cell line. * These breakpoints were defined by G-banding, and SNP array analysis refined them to dup(1)(q23.3q42.12). ** The P16L2 line is listed twice because two independent clones with different aberrations were detected. *** These breakpoints were defined by G-banding, and SNP array analysis refined them to der(18)t(1;18)(q21.2;q21.2).

## Data Availability

The original contributions presented in this study are included in the article/supplementary material. Further inquiries can be directed to the corresponding author.

## Supplementary Materials

The following supporting information can be downloaded at https://www.mdpi.com/article/10.3390/ijms26167728/s1. References [38,39] are cited in the supplementary materials.
