# Supplementary material for: Chromosomal Aberrations in Induced Pluripotent Stem Cells: Identification of Breakpoints in the Large DCC Gene and HIST2 Histone Gene Cluster
Source: Int J Mol Sci. 2025 Aug 10;26(16):7728. doi: 10.3390/ijms26167728 (PMC12387029; doi:10.3390/ijms26167728)
Supplement: Supplementary file 1 [file ijms-26-07728-s001.zip › ijms-3741225-supplementary.pptx]

## Slide 1
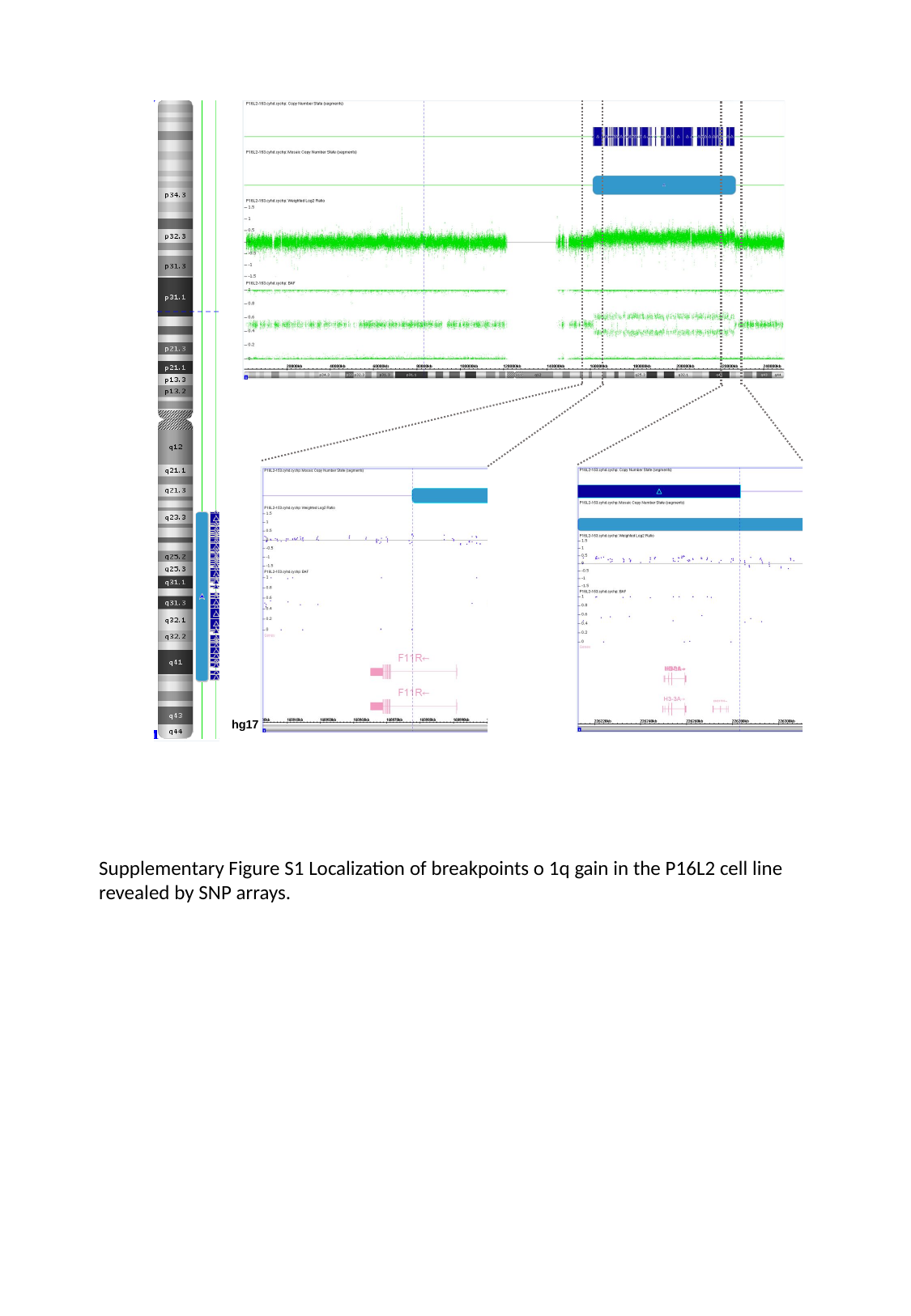

hg17
Supplementary Figure S1 Localization of breakpoints o 1q gain in the P16L2 cell line revealed by SNP arrays.

## Slide 2
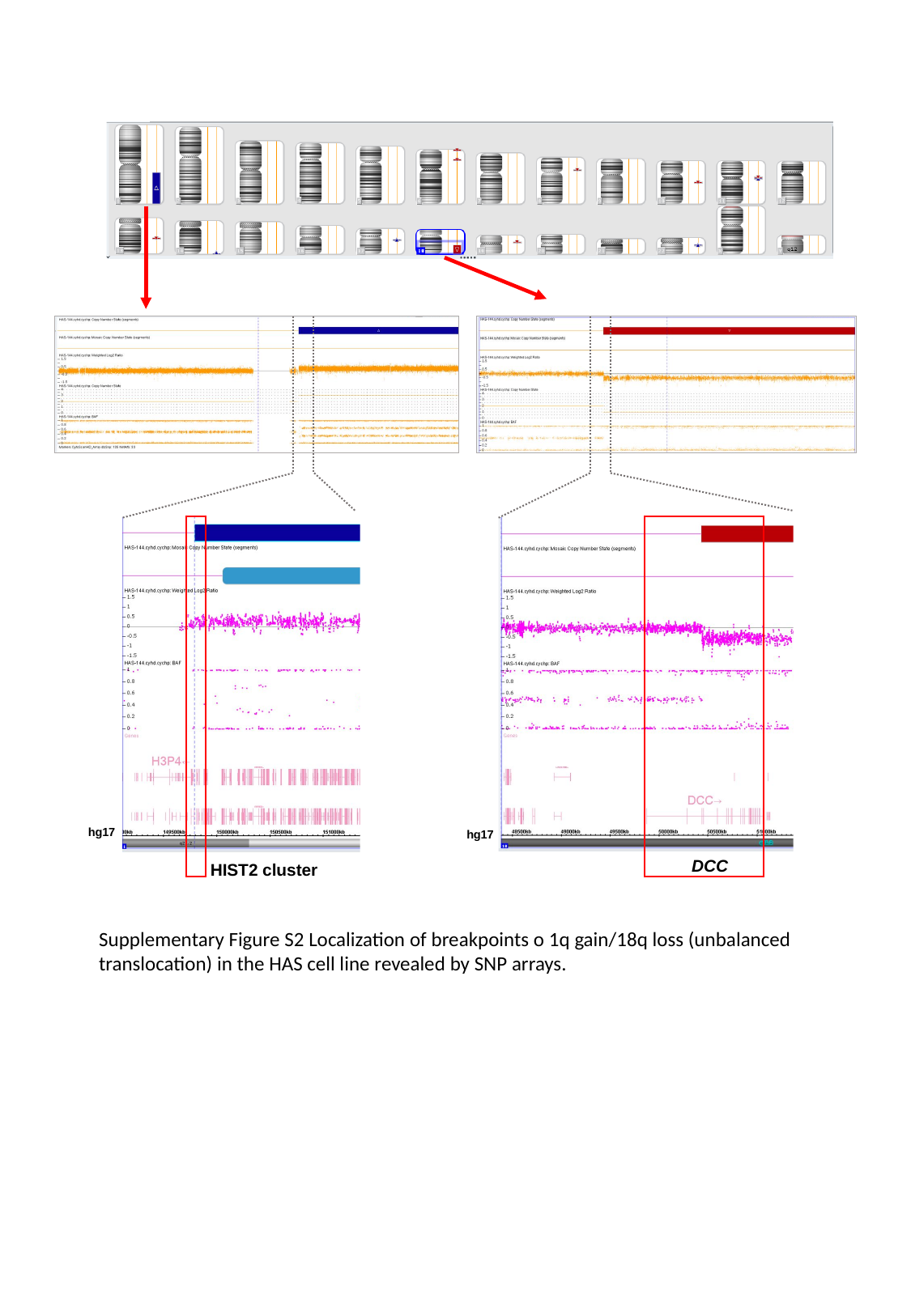

hg17
hg17
DCC
HIST2 cluster
Supplementary Figure S2 Localization of breakpoints o 1q gain/18q loss (unbalanced translocation) in the HAS cell line revealed by SNP arrays.
